# PEPPI: a peptidomic database of human protein isoforms for proteomics experiments

**DOI:** 10.1186/1471-2105-11-S6-S7

**Published:** 2010-10-07

**Authors:** Ao Zhou, Fan Zhang, Jake Y Chen

**Affiliations:** 1 School of Informatics, Indiana University, Indianapolis, IN 46202, USA; 2 Department of Computer and Information Science, School of Science, Purdue University, Indianapolis, IN 46202, USA; 3 Indiana Center for Systems Biology and Personalized Medicine, Indianapolis, IN 46202, USA

## Abstract

**Abstract:**

**Background**

Protein isoform generation, which may derive from alternative splicing, genetic polymorphism, and posttranslational modification, is an essential source of achieving molecular diversity by eukaryotic cells. Previous studies have shown that protein isoforms play critical roles in disease diagnosis, risk assessment, sub-typing, prognosis, and treatment outcome predictions. Understanding the types, presence, and abundance of different protein isoforms in different cellular and physiological conditions is a major task in functional proteomics, and may pave ways to molecular biomarker discovery of human diseases. In tandem mass spectrometry (MS/MS) based proteomics analysis, peptide peaks with exact matches to protein sequence records in the proteomics database may be identified with mass spectrometry (MS) search software. However, due to limited annotation and poor coverage of protein isoforms in proteomics databases, high throughput protein isoform identifications, particularly those arising from alternative splicing and genetic polymorphism, have not been possible.

**Results**

Therefore, we present the PEPtidomics Protein Isoform Database (PEPPI, http://bio.informatics.iupui.edu/peppi), a comprehensive database of computationally-synthesized human peptides that can identify protein isoforms derived from either alternatively spliced mRNA transcripts or SNP variations. We collected genome, pre-mRNA alternative splicing and SNP information from Ensembl. We synthesized *in silico* isoform transcripts that cover all exons and theoretically possible junctions of exons and introns, as well as all their variations derived from known SNPs. With three case studies, we further demonstrated that the database can help researchers discover and characterize new protein isoform biomarkers from experimental proteomics data.

**Conclusions**

We developed a new tool for the proteomics community to characterize protein isoforms from MS-based proteomics experiments. By cataloguing each peptide configurations in the PEPPI database, users can study genetic variations and alternative splicing events at the proteome level. They can also batch-download peptide sequences in FASTA format to search for MS/MS spectra derived from human samples. The database can help generate novel hypotheses on molecular risk factors and molecular mechanisms of complex diseases, leading to identification of potentially highly specific protein isoform biomarkers.

## Background 

Human cells benefit from elaborate mechanisms to modify proteins, creating many protein variants (isoforms), both to increase the diversity of functions and to regulate the activities of proteins. A protein isoform is any of several different forms of the same protein. Different forms of a protein may be produced from related genes such as single-nucleotide polymorphisms (SNPs) or may arise from the same gene by alternative splicing or post-translational modifications (PTM). Alternative splicing and SNPs expands the number of messenger RNAs to about 88,000 mRNA variants during transcription of these genes. About 8% of these protein isoforms are generated from mRNA transcripts affected by alternative splicing or SNPs, whereas over 90% of protein isoforms are created through post-translational modifications (PTMs) after the mRNA is translated into a protein [1]. Recent studies have shown that the identification, analysis and characterization of these individual protein isoforms (Alternative Splicing, SNPs and PTMs) could improve understanding of diseases improve disease diagnosis or interventions [2-11].

Recent advances in clinical proteomics technology, particularly liquid chromatography-coupled tandem mass spectrometry (LC-MS/MS), have enabled biomedical researchers to characterize thousands of proteins in parallel in biological samples[12]. Identifying disease-related protein isoforms using tandem mass spectrometry, therefore, can provide hope for improving both the sensitivity and the specificity of candidate disease biomarkers, because proteomics identification, instead of quantification, of the same set of protein isoforms is often sufficient to distinguish between disease samples and controls.

However, identifying protein isoforms using current MS proteomics search databases and software tools has been challenging, primarily because of the smaller size of known or common alternatively spliced protein isoforms relative to several orders of magnitude larger size of MS search databases, which makes exhaustive novel peptide identification computationally inefficient for routine proteomics studies. Up to 80% of all MS spectra peaks in a typical proteomics experiment may remain uncharacterized when searched against a standard MS database with little protein isoform information. Such standard MS search databases include: the IPI database [13], the NCBI-nr database, and the UniProt knowledge base [14]. These databases integrate more than a dozen public protein and DNA sequence databases into a non-redundant list of both known and predicted protein sequences, with only publicly known splice variant transcripts represented. MS search software such as SEQUEST [15], Mascot [16], X!Tandem [17], and OMSSA [18]. may further allow customized identification of limited types of PTM-derived peptides and proteins. However, these protein sequence databases do not contain information about alternatively spliced transcripts or theoretically possible “mis-spliced” protein isoforms; nor do they contain peptide variants arising from SNPs that result in amino acid changes. Therefore, they are ill-suited for comprehensive protein isoform identification purposes.

Although there are several publicly available alternative splicing mRNA transcript databases and SNP databases including ASTD [19], EID [20,21], ASPicDB [22], ECgene [23], MutDB [24,25], and dbSNP [26], none of these databases can be readily used for identification of novel peptides derived from uncharacterized protein isoforms. Since predictions of gene splicing patterns in all the methods are based on alignments of transcript data (mostly expressed sequence tags, ESTs) to a genomic sequence, some limitations exist in all these methods mostly due to the sequence errors frequently occurring in ESTs and to the repetitive structure of the genome sequence. Moreover, all the databases mentioned contain a rather small set of alternatively spliced peptides because they are either manually curated or literature-based data sets, as well as poor annotation of splice events, which are inadequate for the identification of alternatively spliced protein isoforms. To explore the huge solution space of all possible alternatively spliced combination of exons and potentially coding introns, one must generate virtual peptides exhaustively so that uncharacterized MS spectra can be searched against them. In addition, the database of virtual peptides should be expanded to accommodate the amino acid alterations introduced by each SNP.

In this paper, we describe the development of a **Pep**tideomics Database of **P**rotein **I**soforms (PEPPI), which consists of systematically generated virtual peptides that cover alternative splicing events and known SNP variations, for identifying protein isoforms in large-scale proteomics results. In the PEPPI database, we introduce a peptidomics approach to integrating genome, transcriptome, proteome and SNP information for human proteomics studies. The database contains a comprehensive set of peptides derived from all known annotated human genes in the Genome Reference Consortium Human genome build 37 by generating alternative splicing events and incorporating non-synonymous SNPs. It is the first comprehensive database that can be used to characterize novel protein isoforms derived from alternative splicing and SNP variations in MS spectra. The database has a web user interface that allows its users to query a gene/protein and compare all its above-mentioned types of protein isoforms and associated virtual peptides online. 

## Results 

### Database content

Drawn from Ensembl’s genomic data [27], the PEPPI database contains a comprehensive set of peptides derived from all known human protein-coding genes and was constructed by generating both annotated and hypothetical alternative splicing events and incorporating non-synonymous SNPs. In addition to representing an in-frame peptide for each exonic region (EXON_KB) of human proteins, four types of PEPPI splice junctions are also captured for all possible combinations of each coding sequence of gene: annotated exon-exon junctions (E_E_KB type), hypothetical exon-exon junctions (E_E_TH type), hypothetical exon-intron junctions (E_I_TH type), and hypothetical intron-exon junctions (I_E_TH type). An exonic region or a splice junction is defined as a peptide region. For each peptide region, we also include hypothetical peptides translated with each known non-synonymous SNP. By cataloguing each peptide configurations in the PEPPI database, users can study alternative splicing events such as exon skipping, alternative donor site, alternative accepter site, and intron retention at the proteome level. They can also batch-download the peptide annotation and sequences in FASTA format for MS data searching. The current PEPPI database includes human data only. As of April 2010, it is comprised of 7,848,236 PEPPI peptide entries derived from 23,491 protein-coding genes and 66,384 proteins, incorporating 150,054 non-synonymous SNPs (Table 1).

**Table 1:** Database Content Statistics

Region Type	Regions / Peptides without SNP	Peptides with SNP	All Peptides of the Region Type
EXON_KB	264,599	140,777	405,376
E_E_KB	259,329	146,016	405,345
E_I_TH	400,571	126,678	527,249
I_E_TH	108,754	41,977	150,731
E_E_TH	4,291,289	2,068,246	6,359,535

Total	5,324,542	2,523,694	7,848,236

Mapped Genes: 23,516

Mapped Proteins: 66,384

Non-synonymous SNP: 150,054

A peptide-protein mapping is also captured for comparing the MS search results derived with the PEPPI and conventional protein sequence databases. In total 613,591 peptides are mapped to 66,384 IPI [13] proteins (Table 1).

### General online features 

**Figure 1:**
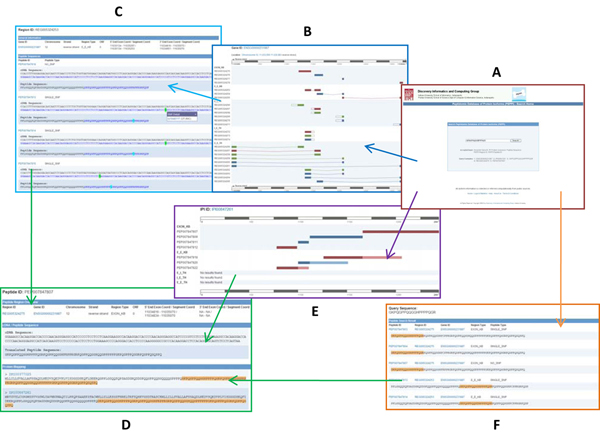
**Web Interface Structure** (A) Search Home: main search page allowing five types of query string: Ensembl gene ID, IPI protein accession number, peptide sequence, PEPPI region ID and peptide ID. (B) Gene View: search result page visualizing peptide regions within a gene. (C) Region View: search result page displaying peptides within a peptide region. (D) Peptide View: PEPPI peptide information page. (E) Protein View: search result page of PEPPI peptides mapped to an IPI protein. (F) Sequence Search: search result page of PEPPI peptides mapped to a query peptide sequence.

In Figure 1, we show the user interfaces of the web-based online version of the PEPPI database. It allows searching by Ensembl Gene ID, gene symbol, UniProt ID, IPI AC, peptide sequence, PEPPI Peptide Region ID and PEPPI Peptide ID.  With the cross-links users can easily link to Ensembl [27], IPI [13], UniProt [28], HAPPI [29] and HPD [30] and get access to much more detailed information about genes, proteins, protein-protein interactions and human pathways. The peptide annotations and sequences are freely available for batch-download in FASTA format on the download page.

### Case studies 

We show three case studies of increasing complexity and biological significance to demonstrate that the database can help researchers discover and characterize new protein isoform biomarkers from experimental proteomics data.

#### Case study 1: browsing PEPPI peptides and relating information based on a query gene 

For users who would like to review all the peptide regions and peptides within a gene of interest, we provide the standard gene search procedure. In this case study we show how to browse the PEPPI peptide regions, PEPPI peptides and related information within gene PRH1. Users will start from the Search Home (Figure 1A), go through the Gene View (Figure 1B), Region View (Figure 1C), and finally navigate to the Peptide View (Figure 1D).

**Figure 2:**
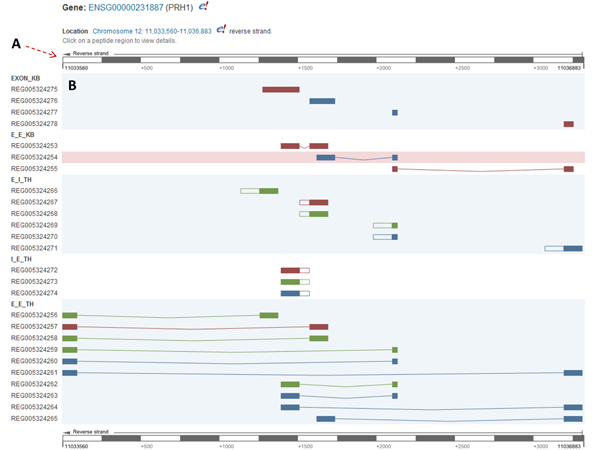
**Gene View** (A) **Gene scale**. Shows the user the chromosome coordinates and strand of the gene. With the gene scale, users can read the approximate position of the peptide regions. (B) **Peptide regions**. Shows the user which five types of peptide regions within current given gene that the peptide belong to. The coloring of the peptide regions indicates the ORF (Red: 0, Green: 1, Blue: 2). The solid bars indicate exons, and the blank bars indicate introns. The curve between two exons means the exons are spliced with each other.

By searching with gene PRH1 in the standard query box provided at the PEPPI database home page, users can retrieve all peptide regions corresponding to this gene (Figure 2). In the Gene View, the PEPPI database visualizes all the peptide regions that can be mapped to this gene. The “Location” section shows this gene is located on chromosome 12, from 11,033,560 bp to 11,036,883 bp. Links to Ensembl are provided on the gene ID and location. A scale of chromosome coordinate is provided on the top and bottom of the visualization. The arrow on the chromosome coordinate scale shows this gene is located on the reverse strand, so the 5’ end of the gene should be the right end. Peptide regions are displayed in five categories, including EXON_KB, E_E_KB, E_I_TH, I_E_TH and E_E_TH. The color of the region indicates the protein translation open reading frame (ORF) of the corresponding cDNA. By clicking on the peptide region REG005324254, the browser will be re-directed to the Region View.

**Figure 3:**
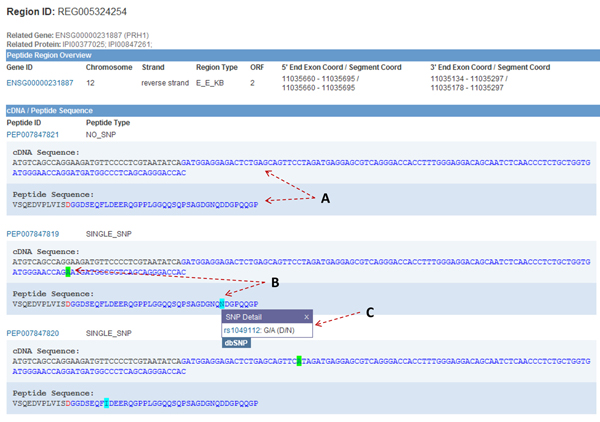
**Region View** (A) The different colors of the cDNA and peptide sequences indicate two different exons, or an exon and an intron. The amino acid letter colored in red overlaps with the splice site. (B) Green and light cyan backgrounds are used to indicate SNP in cDNA and peptide sequences. (C) By clicking on a SNP in sequence, users can see the details of the SNP. A link to the dbSNP database is provided.

The Region View (Figure 3) displays detailed information of the peptide region and the peptide sequences within this region. In the “Peptide Region Overview” section, the exon coordinate is the chromosome coordinate of the source exon, and the segment coordinate is the coordinate of the flanking sequence beside the splice site. The peptide without SNP is displayed on the top of the “cDNA/Peptide Sequence” section, and the peptides with SNPs are displayed below. In the sequences, black and blue are used to color different exons/introns. An amino acid residue overlapping a splice site is colored in red. SNPs are highlighted by green and light cyan. By clicking on the highlighted SNPs, the SNP ID will be shown along with the nucleotide change and amino acid change. A link to the corresponding page in dbSNP is also provided. By clicking on a PEPPI peptide ID, e.g., “PEP007847820”, the browser will be navigated to the Peptide View.

**Figure 4:**
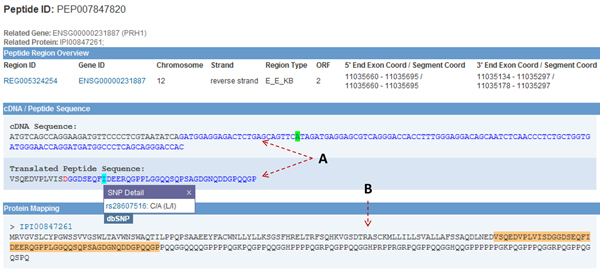
**Peptide View** (A) The cDNA and peptide sequences that the current PEPPI peptide is mapped to. Same color theme is used in the region view. By clicking on SNP in sequence, users can access detailed information of the SNP. (B) In the protein mapping list, the view displays all the proteins mapped to the current PEPPI peptide. The peptide sequence is highlighted from within the protein sequence.

In the Peptide View (Figure 4), detailed information of the peptide region and a peptide-protein mapping is shown in the “Peptide Region Overview” section. The cDNA and peptide sequence is displayed in the same pattern as the Region View. The “Protein Mapping” section lists the proteins mapped to the current peptide. The result shows that IPI00847261 is the only protein mapped to the peptide PEP007847820. The annotation on IPI states that IPI00847261 is one of the protein products of PRH1. Since the peptide PEP007847820 contains a mutant non-synonymous SNP allele, we can infer that the mapped protein IPI00847261 is not the wild-type.

#### Case study 2: identifying genomic origins and alternative splicing events from peptides detected in MS experiments

For users, especially MS proteomics scientists, who want to start the query from a peptide sequence or a protein, we provided a peptide sequence search function (Figure 1F) and the Protein View (Figure 1E). In this case study we demonstrate that the PEPPI database can help identify the genomic origins of peptides detected from MS data, and can help characterize the alternative splicing events related to these peptides.

**Figure 5:**
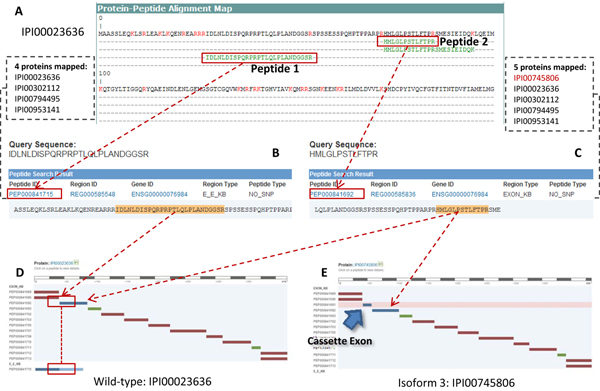
**Identifying The Genomic Origin of MS Detected Peptides and The Relating Alternative Splicing Event** (A) The HIP-2 search result page of protein IPI00023636, displaying the evidence peptides detected in MS experiments. (B) The PEPPI sequence search result page of peptide 1, indicating the query peptide is produced from an exon-exon combination region. The corresponding PEPPI peptide can be mapped to 4 proteins. (C) The sequence search result page of peptide 2, indicating the peptide comes from an exon, and can be mapped to 5 proteins. (D) The search result of the wild-type MP2K7_HUMAN, showing the regions mapped by the peptides. Peptide 1 crosses the splice site of two exons (PEP000841690 and PEP000841692). Peptide 2 is produced from a single exon, PEP000841692. (E) The search result of the 3^rd^ isoform of MP2K7_HUMAN, the protein that is mapped to the peptide 2 but not mapped to peptide 1. That is because the insertion of a cassette exon (PEP000841691) changed the sequence of the protein.

The MS peptides can be derived from Healthy Human Individual's Integrated Plasma Proteome Database (HIP-2) [31] by inputting its protein ID. For this example, by entering “IPI00023636” as the query, a mapping table with several MS peptides identified by the MS data analysis program will be returned (Figure 5A). To identify the genomic region that encodes a specific peptide sequence, we can search the peptide sequence on the PEPPI database’s search home.

As shown in Figure 5B and 5C, peptide 1 is mapped to “PEP000841715”, which is an E_E_KB peptide, and peptide 2 is mapped to “PEP000841692” which is an EXON_KB peptide. This indicates peptide 1 is coded by an exon-exon junction, and peptide 2 is coded by a single exon. 

To study the related alternative splicing events, we then compared the number of proteins which can be mapped to these peptides. By clicking on the peptide ID, we can get access to the proteins mapped to each peptide. We found 4 proteins mapped to peptide 1, and 5 proteins mapped to peptide 2. Interestingly, only one protein (IPI00745806) was differentially mapped. By looking up the protein information in IPI, we found that the proteins involved are five different alternatively spliced isoforms of MP2K7_HUMAN, and IPI00745806 is the third isoform. Therefore, it is likely that only a specific alternative splicing event that takes place is annotated and can be mapped onto the protein sequence IPI00745806.

To verify our suspicion on the existence of the alternative splicing event, we compared the protein-peptide mapping of the wild-type and the third isoform of MP2K7_HUMAN. In the wild-type MP2K7_HUMAN (Figure 5D), “PEP000841715” contains the splice junction of two exons (PEP000841690 and PEP000841692), and peptide 1 just crosses the splice site. Nevertheless, in the MP2K7_HUMAN isoform 3 (Figure 5E), we found a unique cassette exon (PEP000841691) spliced between the two exons, which hampered the coding of sub-sequence mappable to peptide 1. Meanwhile, peptide 2 is only mapped to a single exon (PEP000841692), which exists in all five proteins and unaffected by any splice events. Thus we have confirmed the suspicion that a cassette exon event caused the protein mapping difference between two MS peptides, and have shown the PEPPI database’s ability to help infer alternative splicing events from peptides detected from MS experiments.

#### Case study 3: identifying new peptide isoforms for human 

Human fetal liver can evolve into a major site of embryonic hematopoiesis; therefore, protein profiling may help researchers understand how the interaction between hepatic and hematopoietic systems and the migration of the hematopoietic system during mammalian development take place. We collected four human fetal liver cytoplasm proteome data sets from the human fetal liver project (http://hlpic.hupo.org.cn /dblep). SDS-PAGE with different cross-linking percentages 15%, 10%, and 7.5% was used for protein separation to obtain a full representation of proteins ranging from 5 kDa to more than 300 kDa. After these gels were stained with Colloidal Coomassie Blue R250 and the gel lanes were manually excised from loading position to the bottom of the gel, the extracted peptide mixtures were loaded onto nanoscale LC-ESI-Q-TOF MS or micro-LC-ion trap MS systems for protein identification [32].

In order to show that the PEPPI database can be used to identify additional novel peptide isoforms than the traditional protein database, we downloaded the protein database IPI and created three datasets using the PEPPI database: 1) annotated exonic peptides and exon-exon combinations without SNP (PEPPI_KB), 2) all PEPPI peptides without SNP (PEPPI_without_SNP), and 3) all PEPPI peptides including peptides with SNP (PEPPI_with_SNP). PEPPI_KB consists of peptides of both the EXON_KB and E_E_KB region types without additional SNP permutations; PEPPI_without_SNP consists of peptides of the EXON_KB, E_E_KB, E_I_TH, I_E_TH, and E_E_TH region types without additional SNP permutations; PEPPI_with_SNP consists of peptides of all types, with or without SNPs, in the PEPPI database. We also created four corresponding inverse sequence datasets to evaluate the false discovery rate with a target-decoy search strategy [33]. The four peak list files of human fetal liver from LC-ESI-Q-TOF MS or micro-LC-ion trap MS raw files were searched by OMSSA [18] against the four databases and their four inverse databases in order to compare the results among them. 

OMSSA reports hits ranked by E-value. An E-value for a hit is a score that is the expected number of random hits from a search library to a given spectrum, such that the random hits have an equal or better score than the hit. For example, a hit with an E-value of 1.0 implies that one hit with a score equal to or better than the hit being scored would be expected at random from a sequence library search [18]. The search results with OMSSA can vary substantially with differing search parameters, sequence libraries, and samples [33]. Therefore, we adopted the MS/MS false discovery rate (FDR) instead of E-value as scoring criterion for evaluating the four databases, and this method is based on commonly used scoring methodologies and the target-decoy search strategy [33]. All other OMSSA search parameters [18] for the four databases are the same. To increase identification accuracy, only peptides/proteins with at least two hits of different samples was recognized as true peptides/proteins. 

A comparison of search results against four MS databases, i.e., IPI, PEPPI_KB, PEPPI_without_SNP, and PEPPI_with_SNP, is shown in Table 2. Results are shown only at a commonly used 1% MS/MS FDR for each database. Compared to the traditional IPI database, the elapsed time for PEPPI_KB decreased although the dataset size increased by two and a half times. And with the increase of sizes, the elapsed time increases significantly linearly from PEPPI_KB to PEPPI_without_SNP to PEPPI_with_SNP (Intercept =  8.17021, slope=0.04738 , and adjusted R^2^= 0.9975).

**Table 2:** Summary of Search Results When MS/MS FDR ≤ 0.01

	IPI	PEPPI_KB	PEPPI_without_SNP	PEPPI_with_SNP
Size (M)	47	115.2	1181.1	1769.7
Elapsed Time(h:mm:ss)	0:11:05	0:10:39	1:02:32	1:33:04
MS^2^ FDR	0.01000	0.00992	0.00996	0.00999
Target MS^2^ hits	22685	21473	68137	116363
Decoy MS^2^ hits	114	107	341	584
Peptide FDR	0.01575	0.01527	0.01242	0.01571
Target peptide hits	125	129	159	188
Decoy peptide hits	2	2	2	3
Protein/gene FDR	0.02797	0.02778	0.02439	0.02339
Target protein/gene hits	368/141	142	162	169
Decoy protein/gene hits	5/2	2	2	2
PEPPI FDR		0.01376	0.01283	0.01376
Target PEPPI hits		433	1549	2886
Decoy PEPPI hits		3	10	20

**Figure 6:**
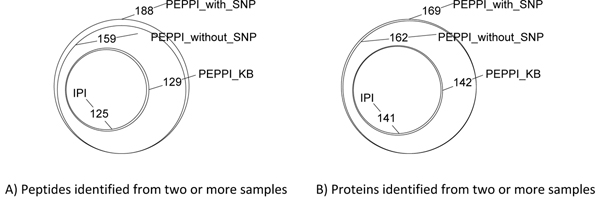
**Overlap of Peptides/Genes Identified by Four Search Databases.** (A) Peptides identified from two or more samples. (B) Proteins identified from two or more samples.

Under the criteria of MS/MS FDR 0.01, the target MS/MS hits markedly increases with the increase of database size, and target peptide hits, target protein hits, and target PEPPI hits all increase while the corresponding FDRs remained approximate. The overlap of genes identified by each database is shown graphically by Venn diagram in Figure 6. The results show that the PEPPI database can be used to identify more peptides/proteins under the same false positive rate than the traditional IPI database.

From the four human fetal liver MS data sets, we identified 63 peptides which mapped to 74 PEPPI peptides and 9 SNP events using PEPPI_with_SNP (See additional file 1). Among the 74 PEPPI peptides, 55 EXON_KB type peptides were also annotated previously in IPI, and 19 peptides were novel peptides uniquely identified with the PEPPI database (13 E_E_KB type peptides, 2 E_E_TH type peptides, 1 I_E_TH type peptide, and 3 E_I_TH type peptides).

The peptide hit matrix shows the number of PEPPI peptides mapped to the peptides detected from the samples, and the number of samples (N) in which the peptide is detected (See additional file 2).

## Discussion and perspectives

We created a comprehensive PEPPI database of both annotated and hypothetical peptides representing human protein isoforms for MS analysis. The PEPPI database made it possible for high-throughput identification of gene variations, exon expression, and alternative splicing events at the proteome level. We also constructed a web-server for searching and visualizing the peptides. With the user-friendly interface and powerful search functions, users can easily study the alternative splicing events and gene variations related to any gene, protein, or peptide sequence of interest.

A comparison between the PEPPI database and conventional MS methods is shown in Table 3. An MS approach with the PEPPI database uses the same samples, equipments and analysis software as a conventional MS approach. To use the PEPPI database, users just need to set the PEPPI database or a subset of the PEPPI database as the user defined sequence database in the MS search software. With the PEPPI database, users can gain information on the expression of exons, alternative splicing events, SNPs, and protein existence from the proteome, while the conventional MS approach can only derive the protein existence information. Users can opt to use different subsets of the PEPPI database for different study purposes. The computational cost of adopting the PEPPI database approach over a conventional approach is kept low, due to the use of computing cycles without human intervention.

**Table 3:** Comparison with Conventional MS Methods

	MS with PEPPI	Conventional MS Methods
**Sample **	Same as right	Ordinary proteome samples
**Spectrum**	Same as right	Ordinary MS equipments
**Software**	Same as right	SEQUEST, Mascot, OMSSA etc.
**Sequence Database**	PEPPI or subset of PEPPI	Conventional protein sequence databases (IPI, UniProtKB, e.g.)
**Detection Ability**	1. Exonic region 2. Exon-Exon combinations (annotated transcripts) 3. Exon-Intron combinations 4. Intron-Exon combinations 5. Hypothetical Exon-Exon combinations 6. SNP peptides	Only proteins
**Configuration**	User configurable	Pre-defined by database producer
**Cost **	Same as right	Not very expensive

Since the database incorporated a large number of hypothetical peptides, it is possible that the search result contains false positives due to the noise. To solve this problem, we will design an optimized routine for MS data analysis. For example, users may analyze several samples at one time, and only keep the peptides detected in more than one sample. On the other hand, a recently published article [34] reported tissue dependent splicing patterns, which make it possible to generate tissue specific PEPPI peptides and reduce the chance of incorporating false positives.

In the future, we plan to enhance the user interface of the web application such as replacing the static download page with an interactive batch-download interface, where users can specify the peptide type, gene IDs and protein IDs. We also plan to improve the performance of peptide sequence search with parallel computing and a more powerful database server.

## Methods 

### Genome data source

**Figure 7:**
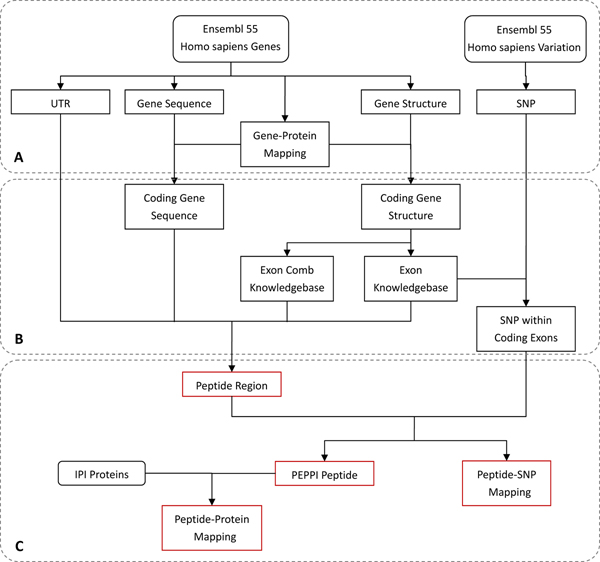
**Data Generation Process.** The whole data generation process was divided into three steps: (A) deriving the genome data from Ensembl; (B) pre-processing of the data to select protein-coding genes and SNPs within coding exons; and (C) generation of peptide regions and PEPPI peptides. The result datasets are colored in red.

The PEPPI peptides were generated from the human genome. The source genome data was downloaded from Ensembl Version 55 [27] with BioMart[35]. As shown in Figure 7A, four tables, including Un-Translated Region (UTR), Gene Sequence, Gene-Protein Mapping and Gene Structure, were pulled from the Ensembl Homo sapiens Genes Dataset. The UTR table describes the coordinates for all transcript UTRs. The Gene Sequence table contains chromosome coordinates and sequences in FASTA format. The Gene Structure table contains the exon annotation information, including Exon ID, Gene ID, Transcript ID, as well as the genome coordinate and translation phase of the exons. The SNP table was derived from the Ensembl Homo sapiens Variation Dataset, and contains the SNP’s chromosome coordinate and nucleotide shift. The PEPPI database incorporated 44,285 genes and 16,489,577 SNPs.

### Data pre-processing

A data pre-processing procedure (Figure 7B) was implemented to remove non-coding genes and SNPs which are not in exonic regions. 

Firstly we imported all the source tables into a SQLite3 database with the SQLite3 command-line interface, and then we used SQL statements to remove non-coding genes. The Gene-Protein Mapping was utilized as a filter, and genes not mapped to proteins were considered as non-coding genes. The Coding Gene Sequence and Coding Gene Structure table was derived after filtering, and 21,351 protein-coding genes were captured. 

Then we compiled a C program with the SQLite3 library to extract the annotated transcription information from the Coding Gene Structure table, and produced two tables. The Exon Knowledgebase table describes all the protein-coding exons, and the Exon Comb Knowledgebase table describes all the exon-exon combinations found in the annotated transcripts. Then the SNP table was searched against the Exon Knowledgebase table, and 390,539 SNPs within the annotated coding exons were retrieved for peptide generation.

### Peptide region generation

We compiled a pipeline program with C and the SQLite3 library to generate peptide regions (Figure 7C). The program first generated the wild-type cDNA sequences of the peptide region, and then translated the cDNA sequences into peptides. The derived peptides were estimated by the program according to a set of protocols, and un-qualified peptides and the corresponding region were discarded. Different cDNA generation procedures and peptide estimation protocols were implemented on different types of peptide regions (Table 4).

**Table 4:** Data Generation Protocols

	cDNA Sequence Length	Open Reading Frame (ORF)	Stop Codon Toleration
EXON_KB	Whole Length	Annotated ORF Only	Not Tolerated
E_E_KB	≤ 240 bp	Annotated ORF Only	Not Tolerated
E_I_TH	≤ 240 bp	All 3 ORFs	In 3’ end segment
I_E_TH	≤ 240 bp	All 3 ORFs	Not Tolerated
E_E_TH	≤ 240 bp	All 3 ORFs	In 3’ end segment

We limited the lengths of the combination transcripts to 240 bp, with a flanking sequence of 120 bp on each side of the splice site.

For the EXON_KB type, the chromosome coordinate of the exons were derived directly from the Exon Knowledgebase table, and the whole length of the exon cDNA sequence was captured from the gene sequence. Then the exon’s cDNA sequences were translated into peptides according to the annotated ORF. In the peptide estimation process, if a stop codon existed anywhere except the end of the peptide, the corresponding region was considered invalid and was discarded.

Similar to the EXON_KB type, the chromosome coordinates of the two exons in the E_E_KB type were derived directly from the Exon Comb Knowledgebase table. Then the derived cDNA sequences were translated into peptides according to the annotated ORF of the exon on the 5’ end. The same peptide estimation protocol used with the EXON_KB type was applied to the E_E_KB type.

For the E_I_TH and I_E_TH type, the program derived the chromosome coordinates of exons from the Exon Knowledgebase table and spliced them with the adjacent introns. The cDNA flanking sequence on both side of the splice site was limited to 120 nucleotides. If the exon/intron is shorter than 120 nucleotides, the program will pull out the actual sequence. This limit was set according to the longest peptide in the HIP-2 database [31], which had a length of 80 amino acids and corresponded to 240 nucleotides in the cDNA sequence. This represents the length of the longest peptide that can be identified from an MS experiment. In this way we made sure that any MS identified peptide that crosses the splice site can be captured by the PEPPI database. In the peptide estimation process, a stop codon is tolerated in the intron of E_I_TH, but not tolerated anywhere expect the 3’ end in I_E_TH.

When producing the E_E_TH type of peptide regions, all possible exon-exon combinations were generated and searched against the Exon Comb table. Any combinations that cannot be found in the Exon Comb table were captured as an E_E_TH type candidate. Then each E_E_TH type cDNA was translated in all 3 ORFs, and if a stop codon was found in the 5’ end exon, the peptide was discarded. Note if more than one E_E_TH peptide derived from the 3 ORFs were considered valid, then a peptide region was created for each ORF.

### PEPPI peptide generation

After the generation of peptide region, PEPPI peptides were produced by inserting non-synonymous SNPs into the wild-type peptide of the corresponding region (Figure 7C). All the non-synonymous SNPs within a peptide region were first captured in a list, and then inserted into the wild-type cDNA according to their chromosome coordinates. Each cDNA sequence with SNP was then translated into peptides. The peptides were then estimated according to the peptide estimation protocol of its own region type, and invalid ones were discarded. During peptide generation, a table of Peptide-SNP Mappings was also generated. The wild type peptides were also deposited in the PEPPI Peptide table. 

### Online PEPPI server design

The online version of PEPPI database is a typical 3-tier web application, with a MySQL database at the backend database service layer, Apache/PHP server scripts to the middleware application web server layer, and CSS driven web pages presented on the browser. The Javascript library uuCanvas (http://uupaa-js-spinoff.googlecode.com/svn/trunk/uuCanvas.js/) is used to render the real-time data visualizations in the gene view and the protein view. 

**Figure 8:**
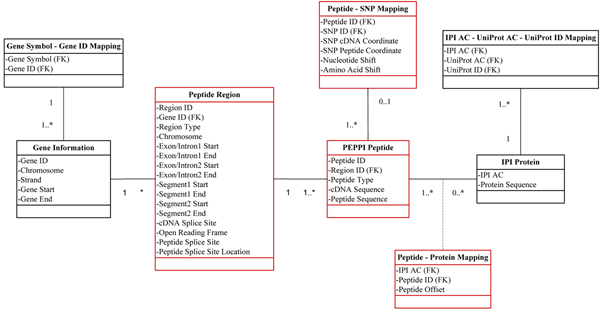
**The UML of Database Backend** The datasets derived by the data generation pipeline are colored in red, and the datasets derived from other databases are colored in black.

The result tables derived from the data generation step were imported into the MySQL database (Figure 8). The chromosome coordinate information was deposited in the Peptide Region table, and the sequence information was deposited in the PEPPI Peptide table. The ID mapping tables for genes and proteins enable users to query the database with different IDs. 

## Authors' contributions

JYC conceived the initial work, designed the method for the database construction. AZ implemented the design, generated the datasets, and developed the database backend and the web-based interface from the generated datasets. FZ collected and analyzed the MS data, performed the statistical analyses of the case studies. All authors are involved in the drafting and revisions of the manuscript.

## Competing interests

The authors declare that they have no competing interests.

## Supplementary Material

Additional File 1 Click here for file

Additional File 2 N: number of samples in which the peptide is detected The 2nd to 9th columns are 8 human fetal liver samples marked by Pride accession numbers. The digits in the 8 columns represent the numbers of PEPPI peptides mapped to the peptides detected from the samples. Click here for file
