# Supplementary material for: PEPPI: a peptidomic database of human protein isoforms for proteomics experiments
Source: BMC Bioinformatics. 2010 Oct 7;11(Suppl 6):S7. doi: 10.1186/1471-2105-11-S6-S7 (PMC3026381; doi:10.1186/1471-2105-11-S6-S7)
Supplement: Additional File 1 [file 1471-2105-11-S6-S7-S1.doc]

Additional File 1 - 63 peptides identified by PEPPI database.

| Peptide | peppi | SNP | Gene | Splicing | Left_Start | Left_End | Right_Start | Right_End | FDR | Mass | Charge | Theo Mass |
| --- | --- | --- | --- | --- | --- | --- | --- | --- | --- | --- | --- | --- |
| AGEYGAEALER | PEP006194756 | rs33943087(A/G) | ENSG00000188536 | E_E_KB | 222875 | 223006 | 223124 | 223328 | 0.0032 | 1165.19 | 2 | 1164.54 |
| APVPTGEVYFADSFDR | PEP003042584 |  | ENSG00000127022 | EXON_KB | 179133259 | 179133332 |  |  | 0.0073 | 1769.79 | 2 | 1769.83 |
| ASSVVVSGTPIR | PEP001086304 |  | ENSG00000103876 | EXON_KB | 80460394 | 80460491 |  |  | 0.0100 | 1173.11 | 2 | 1171.66 |
| ATSFLLALEPELEAR | PEP004603386 |  | ENSG00000163902 | EXON_KB | 128369383 | 128369719 |  |  | 0.0031 | 1660.01 | 2 | 1658.89 |
| AVFPSIVGR | PEP006209453 | rs11549244(C/T) | ENSG00000184009 | EXON_KB | 79479258 | 79479386 |  |  | 0.0031 | 945.881 | 2 | 944.543 |
| AVFVDLEPTVLDEVR | PEP006626718 | rs36215077(G/C) | ENSG00000198033 | E_E_KB | 19753481 | 19753703 | 19752386 | 19752534 | 0.0067 | 1701.74 | 2 | 1700.9 |
| AVFVNLEPTVIDEVR | PEP002507811 | rs11546624(G/A) | ENSG00000123416 | E_E_KB | 49523283 | 49523505 | 49523025 | 49523173 | 0.0066 | 1700.66 | 2 | 1699.91 |
| AVNTLNEALEFAK | PEP000166109 |  | ENSG00000021826 | E_E_KB | 211512587 | 211512781 | 211513197 | 211513264 | 0.0088 | 1418.72 | 2 | 1418.74 |
| DLLDDLKSELTGK | PEP004712877 |  | ENSG00000164111 | EXON_KB | 122604519 | 122604632 |  |  | 0.0062 | 1445.69 | 2 | 1445.76 |
| DPQLVPILIEAAR | PEP003888536 |  | ENSG00000147383 | EXON_KB | 152034363 | 152034505 |  |  | 0.0036 | 1435.05 | 2 | 1433.83 |
| EGDVLTLLESER | PEP007510716 |  | ENSG00000227097 | EXON_KB | 82400601 | 82400810 |  |  | 0.0090 | 1359.8 | 2 | 1359.69 |
| EGDVLTLLESER | PEP007749514 |  | ENSG00000233927 | EXON_KB | 8386837 | 8386975 |  |  | 0.0090 | 1359.8 | 2 | 1359.69 |
| ELFSNLQEFAGPSGK | PEP006264479 |  | ENSG00000185432 | EXON_KB | 51318822 | 51319100 |  |  | 0.0035 | 1622.75 | 2 | 1622.79 |
| ETTIQGLDGLSER | PEP003467192 |  | ENSG00000136872 | E_E_KB | 104190751 | 104190805 | 104189764 | 104189924 | 0.0066 | 1417.67 | 2 | 1417.71 |
| FGGLLLTEKPIVLK | PEP004835823 |  | ENSG00000160870 | EXON_KB | 99302615 | 99303218 |  |  | 0.0032 | 1526.87 | 2 | 1526.94 |
| FLEQQDQVLQTK | PEP005639485 | rs61726455(A/G) | ENSG00000172867 | EXON_KB | 53044123 | 53044337 |  |  | 0.0089 | 1477.5 | 2 | 1475.76 |
| FPGQLNADLR | PEP001260206 |  | ENSG00000101162 | EXON_KB | 57598760 | 57601709 |  |  | 0.0085 | 1131.11 | 2 | 1129.59 |
| FPGQLNADLR | PEP001635184 |  | ENSG00000104833 | EXON_KB | 6494331 | 6496232 |  |  | 0.0085 | 1131.11 | 2 | 1129.59 |
| FPGQLNADLR | PEP003177389 |  | ENSG00000137267 | EXON_KB | 3153919 | 3155157 |  |  | 0.0085 | 1131.11 | 2 | 1129.59 |
| FPGQLNADLR | PEP003198554 | rs1054331(G/C) | ENSG00000137285 | EXON_KB | 3224495 | 3226045 |  |  | 0.0085 | 1131.11 | 2 | 1129.59 |
| FVTVQTISGTGALR | PEP002709222 |  | ENSG00000125166 | EXON_KB | 58753100 | 58753159 |  |  | 0.0100 | 1448.73 | 2 | 1448.8 |
| GALQNIIPASTGAAK | PEP001993065 | rs11549348(G/C) | ENSG00000111640 | E_E_TH | 6643920 | 6644027 | 6646750 | 6647162 | 0.0066 | 1411.99 | 2 | 1410.78 |
| GLGTDEDTIIDIITHR | PEP006091881 |  | ENSG00000197043 | E_E_KB | 150503847 | 150503928 | 150502478 | 150502572 | 0.0033 | 1767.84 | 2 | 1767.9 |
| GLSEDTTEETLK | PEP002153670 |  | ENSG00000115053 | EXON_KB | 232320721 | 232320847 |  |  | 0.0025 | 1322.28 | 2 | 1321.63 |
| GTEDFIVESLDASFR | PEP002703216 |  | ENSG00000124783 | EXON_KB | 7301543 | 7301805 |  |  | 0.0071 | 1684.73 | 2 | 1684.79 |
| GTLYIIKLSADIR | PEP002266542 |  | ENSG00000115593 | I_E_TH |  |  | 88407890 | 88408058 | 0.0046 | 1463.68 | 2 | 1461.86 |
| GTVTDFPGFDER | PEP004712880 |  | ENSG00000164111 | EXON_KB | 122607443 | 122607527 |  |  | 0.0031 | 1341.46 | 2 | 1339.6 |
| IFTSIGEDYDER | PEP005147259 |  | ENSG00000167085 | EXON_KB | 47486693 | 47486835 |  |  | 0.0000 | 1444.87 | 2 | 1443.65 |
| IITLTGPTNAIFK | PEP005100585 |  | ENSG00000169564 | EXON_KB | 70314585 | 70316332 |  |  | 0.0039 | 1389.78 | 2 | 1387.81 |
| ILGVGPDDPDLVR | PEP004526503 |  | ENSG00000160285 | EXON_KB | 47641768 | 47641889 |  |  | 0.0014 | 1366.67 | 2 | 1364.73 |
| IPNPDFFEDLEPFR | PEP003042599 |  | ENSG00000127022 | EXON_KB | 179149805 | 179150020 |  |  | 0.0066 | 1734.8 | 2 | 1734.83 |
| IWHHTFYNELR | PEP000663301 |  | ENSG00000075624 | EXON_KB | 5568847 | 5569031 |  |  | 0.0062 | 1514.73 | 2 | 1514.74 |
| KIPNPDFFEDLEPFR | PEP003042599 |  | ENSG00000127022 | EXON_KB | 179149805 | 179150020 |  |  | 0.0098 | 1862.9 | 2 | 1862.92 |
| KPEEVDDEVFYSPR | PEP000367628 |  | ENSG00000036473 | EXON_KB | 38271115 | 38271252 |  |  | 0.0072 | 1708.73 | 2 | 1708.8 |
| LASDLLEWIR | PEP000700443 |  | ENSG00000072110 | E_E_KB | 69360372 | 69360464 | 69358770 | 69359000 | 0.0062 | 1214.65 | 2 | 1214.67 |
| LASDLLEWIR | PEP002700953 |  | ENSG00000130402 | E_E_KB | 39205109 | 39205201 | 39207726 | 39207932 | 0.0062 | 1214.65 | 2 | 1214.67 |
| LATQSNEITIPVTFESR | PEP001796472 |  | ENSG00000106211 | EXON_KB | 75933301 | 75933609 |  |  | 0.0055 | 1904.91 | 2 | 1904.99 |
| LAVDEEENADNNTK | PEP000643567 |  | ENSG00000072274 | EXON_KB | 195802030 | 195802231 |  |  | 0.0050 | 1560.65 | 2 | 1560.69 |
| LDETDDPDDYGDR | PEP000150404 |  | ENSG00000014216 | EXON_KB | 64972154 | 64972329 |  |  | 0.0081 | 1524.78 | 2 | 1524.59 |
| LGANSLLDLVVFGR | PEP007587517 | rs6898538(A/G) | ENSG00000232961 | E_I_TH | 1593228 | 1593399 |  |  | 0.0081 | 1473.02 | 2 | 1472.83 |
| LGGPEAGLGEYLFER | PEP000526191 |  | ENSG00000087086 | EXON_KB | 49469840 | 49470135 |  |  | 0.0072 | 1606.99 | 2 | 1606.8 |
| LLDNWDSVTSTFSK | PEP002177464 |  | ENSG00000118137 | EXON_KB | 116706469 | 116707127 |  |  | 0.0100 | 1611.71 | 2 | 1611.78 |
| LLSGEDVGQDEGATR | PEP000839802 |  | ENSG00000070182 | EXON_KB | 65259715 | 65260585 |  |  | 0.0094 | 1545.73 | 2 | 1545.73 |
| LPLQDVYK | PEP001557134 |  | ENSG00000101210 | E_I_TH | 62124490 | 62124640 |  |  | 0.0028 | 974.937 | 2 | 974.544 |
| LPLQDVYK | PEP001557050 |  | ENSG00000101210 | EXON_KB | 62124490 | 62124640 |  |  | 0.0028 | 974.937 | 2 | 974.544 |
| LPLQDVYK | PEP004163143 |  | ENSG00000156508 | EXON_KB | 74228421 | 74228571 |  |  | 0.0028 | 974.937 | 2 | 974.544 |
| LQEAAELEAVELPVPIR | PEP000044823 |  | ENSG00000004939 | EXON_KB | 42336531 | 42336712 |  |  | 0.0081 | 1876.24 | 2 | 1876.03 |
| LRVDPVNFK | PEP006194445 | rs41515552(G/A) | ENSG00000188536 | EXON_KB | 223124 | 223328 |  |  | 0.0098 | 1086.58 | 2 | 1086.62 |
| LSESHPDATEDLQR | PEP004546571 |  | ENSG00000163554 | EXON_KB | 158617329 | 158617510 |  |  | 0.0081 | 1597.72 | 2 | 1596.74 |
| LVNVVLGAHNVR | PEP006276907 |  | ENSG00000196415 | EXON_KB | 843893 | 844034 |  |  | 0.0032 | 1291.44 | 2 | 1289.76 |
| NIEDVIAQGIGK | PEP006028640 |  | ENSG00000177600 | E_E_KB | 811597 | 811645 | 812535 | 812633 | 0.0057 | 1255.63 | 2 | 1255.68 |
| NILGGTVFR | PEP003130712 |  | ENSG00000138413 | EXON_KB | 209113093 | 209113384 |  |  | 0.0062 | 977.345 | 2 | 975.549 |
| QEYDESGPSIVHR | PEP000663267 |  | ENSG00000075624 | EXON_KB | 5566787 | 5567522 |  |  | 0.0072 | 1515.61 | 2 | 1515.7 |
| QEYDESGPSIVHR | PEP006209424 |  | ENSG00000184009 | EXON_KB | 79476999 | 79477859 |  |  | 0.0072 | 1515.61 | 2 | 1515.7 |
| QEYDESGPSIVHR | PEP006500247 |  | ENSG00000188219 | EXON_KB | 132020928 | 132022250 |  |  | 0.0072 | 1515.61 | 2 | 1515.7 |
| QITLNDLPVGR | PEP002213772 |  | ENSG00000123131 | EXON_KB | 23700513 | 23700643 |  |  | 0.0046 | 1225.31 | 2 | 1224.68 |
| QNQIAVDEIR | PEP000031946 |  | ENSG00000005381 | EXON_KB | 56350109 | 56350279 |  |  | 0.0088 | 1186.55 | 2 | 1184.62 |
| RLFEGNALLR | PEP005143967 |  | ENSG00000170889 | E_E_KB | 54705355 | 54705477 | 54710144 | 54711498 | 0.0090 | 1187.7 | 2 | 1187.68 |
| RLSEDYGVLK | PEP005474171 |  | ENSG00000167815 | EXON_KB | 12910991 | 12911113 |  |  | 0.0072 | 1178.61 | 2 | 1178.63 |
| SFAAVIQALDGEMR | PEP000671849 |  | ENSG00000079459 | E_E_KB | 11666303 | 11666400 | 11667176 | 11667359 | 0.0025 | 1506.71 | 2 | 1506.75 |
| SLLEGEGSSGGGGR | PEP006172793 |  | ENSG00000186395 | E_E_KB | 38975769 | 38975986 | 38975039 | 38975413 | 0.0031 | 1262.46 | 2 | 1261.59 |
| SNPEDQILYQTER | PEP001630051 |  | ENSG00000110917 | EXON_KB | 121131894 | 121132072 |  |  | 0.0062 | 1591.97 | 2 | 1591.75 |
| SNPEDQILYQTER | PEP001630055 |  | ENSG00000110917 | E_E_KB | 121124949 | 121125334 | 121131894 | 121132072 | 0.0062 | 1591.97 | 2 | 1591.75 |
| SQIHDIVLVGGSTR | PEP001592926 |  | ENSG00000109971 | EXON_KB | 122930181 | 122930736 |  |  | 0.0067 | 1480.77 | 2 | 1480.8 |
| SYELPDGQVITIGNER | PEP000664177 |  | ENSG00000075624 | E_I_TH | 5567912 | 5568194 |  |  | 0.0049 | 1789.73 | 2 | 1789.89 |
| SYELPDGQVITIGNER | PEP001946883 |  | ENSG00000107796 | EXON_KB | 90699264 | 90699455 |  |  | 0.0049 | 1789.73 | 2 | 1789.89 |
| TGAIVDVPVGEELLGR | PEP004285208 |  | ENSG00000152234 | EXON_KB | 43669789 | 43669962 |  |  | 0.0072 | 1624.07 | 2 | 1623.88 |
| THLAPYSDELR | PEP002177456 |  | ENSG00000118137 | EXON_KB | 116706467 | 116707127 |  |  | 0.0097 | 1300.62 | 2 | 1300.64 |
| TPAQYDASELK | PEP005624473 |  | ENSG00000182718 | EXON_KB | 60653140 | 60653253 |  |  | 0.0025 | 1221.54 | 2 | 1221.59 |
| VEYHFLSPYVSPK | PEP000643542 |  | ENSG00000072274 | EXON_KB | 195776156 | 195779055 |  |  | 0.0082 | 1564.75 | 2 | 1564.79 |
| VGGVQSLGGTGALR | PEP002477459 |  | ENSG00000120053 | EXON_KB | 101166483 | 101166606 |  |  | 0.0023 | 1271.8 | 2 | 1270.7 |
| VLSGDLGQLPTGIR | PEP001210793 |  | ENSG00000100889 | EXON_KB | 24566101 | 24566346 |  |  | 0.0090 | 1424.95 | 2 | 1424.8 |
| VTQWAEER | PEP003255754 |  | ENSG00000137177 | E_E_TH | 17817251 | 17817431 | 17790103 | 17790141 | 0.0067 | 1019.25 | 2 | 1017.49 |
| YLSYTLNPDLIR | PEP005271414 |  | ENSG00000166825 | EXON_KB | 90334184 | 90334324 |  |  | 0.0000 | 1467.65 | 2 | 1466.78 |
