# Supplementary material for: PEPPI: a peptidomic database of human protein isoforms for proteomics experiments
Source: BMC Bioinformatics. 2010 Oct 7;11(Suppl 6):S7. doi: 10.1186/1471-2105-11-S6-S7 (PMC3026381; doi:10.1186/1471-2105-11-S6-S7)
Supplement: Additional File 2 — N: number of samples in which the peptide is detected The 2nd to 9th columns are 8 human fetal liver samples marked by Pride accession numbers. The digits in the 8 columns represent the numbers of PEPPI peptides mapped to the peptides detected from the samples. [file 1471-2105-11-S6-S7-S2.doc]

Additional File 2 – Peptide hit matrix

|  | PRIDE1780 | PRIDE1781 | PRIDE1783 | PRIDE1784 | PRIDE1785 | PRIDE1786 | PRIDE1848 | PRIDE1849 | N |
| --- | --- | --- | --- | --- | --- | --- | --- | --- | --- |
| AGEYGAEALER | 1 | 0 | 0 | 0 | 0 | 0 | 0 | 7 | 2 |
| APVPTGEVYFADSFDR | 0 | 0 | 5 | 2 | 0 | 2 | 0 | 0 | 3 |
| ASSVVVSGTPIR | 0 | 2 | 0 | 0 | 0 | 0 | 2 | 0 | 2 |
| ATSFLLALEPELEAR | 1 | 0 | 0 | 0 | 1 | 0 | 0 | 0 | 2 |
| AVFPSIVGR | 0 | 1 | 0 | 0 | 0 | 0 | 2 | 2 | 3 |
| AVFVDLEPTVLDEVR | 1 | 1 | 1 | 0 | 1 | 0 | 1 | 0 | 5 |
| AVFVNLEPTVIDEVR | 3 | 4 | 0 | 0 | 5 | 0 | 3 | 0 | 4 |
| AVNTLNEALEFAK | 0 | 0 | 0 | 4 | 0 | 0 | 25 | 0 | 2 |
| DLLDDLKSELTGK | 0 | 0 | 0 | 2 | 0 | 0 | 0 | 4 | 2 |
| DPQLVPILIEAAR | 0 | 1 | 1 | 0 | 1 | 0 | 0 | 0 | 3 |
| EGDVLTLLESER | 0 | 0 | 0 | 0 | 1 | 0 | 0 | 1 | 2 |
| ELFSNLQEFAGPSGK | 0 | 0 | 1 | 1 | 0 | 0 | 0 | 0 | 2 |
| ETTIQGLDGLSER | 0 | 0 | 0 | 2 | 0 | 0 | 3 | 0 | 2 |
| FGGLLLTEKPIVLK | 0 | 0 | 5 | 0 | 0 | 1 | 0 | 0 | 2 |
| FLEQQDQVLQTK | 7 | 4 | 0 | 0 | 0 | 2 | 1 | 0 | 4 |
| FPGQLNADLR | 0 | 1 | 2 | 1 | 2 | 0 | 0 | 0 | 4 |
| FVTVQTISGTGALR | 0 | 0 | 0 | 0 | 0 | 1 | 4 | 0 | 2 |
| GALQNIIPASTGAAK | 0 | 0 | 4 | 3 | 0 | 1 | 3 | 0 | 4 |
| GLGTDEDTIIDIITHR | 0 | 0 | 3 | 3 | 0 | 0 | 0 | 0 | 2 |
| GLSEDTTEETLK | 0 | 0 | 0 | 0 | 0 | 0 | 6 | 1 | 2 |
| GTEDFIVESLDASFR | 0 | 0 | 1 | 1 | 0 | 0 | 0 | 0 | 2 |
| GTLYIIKLSADIR | 0 | 1 | 0 | 0 | 0 | 0 | 0 | 1 | 2 |
| GTVTDFPGFDER | 1 | 3 | 0 | 0 | 0 | 0 | 0 | 0 | 2 |
| IFTSIGEDYDER | 1 | 1 | 0 | 0 | 1 | 4 | 0 | 0 | 4 |
| IITLTGPTNAIFK | 0 | 0 | 0 | 0 | 0 | 0 | 1 | 1 | 2 |
| ILGVGPDDPDLVR | 0 | 3 | 0 | 0 | 1 | 3 | 0 | 0 | 3 |
| IPNPDFFEDLEPFR | 0 | 0 | 5 | 2 | 0 | 0 | 0 | 0 | 2 |
| IWHHTFYNELR | 0 | 0 | 0 | 2 | 0 | 0 | 3 | 0 | 2 |
| KIPNPDFFEDLEPFR | 0 | 0 | 5 | 0 | 0 | 2 | 0 | 0 | 2 |
| KPEEVDDEVFYSPR | 0 | 0 | 1 | 0 | 0 | 0 | 2 | 0 | 2 |
| LASDLLEWIR | 0 | 0 | 0 | 1 | 0 | 0 | 17 | 0 | 2 |
| LATQSNEITIPVTFESR | 0 | 0 | 0 | 2 | 0 | 1 | 0 | 0 | 2 |
| LAVDEEENADNNTK | 0 | 0 | 4 | 5 | 0 | 1 | 0 | 0 | 3 |
| LDETDDPDDYGDR | 0 | 1 | 0 | 0 | 0 | 0 | 1 | 0 | 2 |
| LGANSLLDLVVFGR | 0 | 0 | 0 | 1 | 1 | 0 | 0 | 0 | 2 |
| LGGPEAGLGEYLFER | 0 | 0 | 4 | 2 | 2 | 0 | 0 | 0 | 3 |
| LLDNWDSVTSTFSK | 0 | 0 | 0 | 2 | 0 | 0 | 0 | 5 | 2 |
| LLSGEDVGQDEGATR | 0 | 0 | 0 | 8 | 0 | 0 | 1 | 0 | 2 |
| LPLQDVYK | 0 | 0 | 0 | 0 | 0 | 0 | 4 | 3 | 2 |
| LQEAAELEAVELPVPIR | 0 | 0 | 0 | 4 | 2 | 0 | 0 | 0 | 2 |
| LRVDPVNFK | 0 | 0 | 3 | 0 | 0 | 0 | 0 | 7 | 2 |
| LSESHPDATEDLQR | 0 | 2 | 0 | 0 | 0 | 0 | 3 | 0 | 2 |
| LVNVVLGAHNVR | 1 | 1 | 0 | 0 | 0 | 0 | 0 | 0 | 2 |
| NIEDVIAQGIGK | 0 | 0 | 2 | 0 | 0 | 0 | 0 | 1 | 2 |
| NILGGTVFR | 0 | 1 | 0 | 0 | 0 | 0 | 6 | 0 | 2 |
| QEYDESGPSIVHR | 0 | 0 | 0 | 0 | 0 | 2 | 4 | 3 | 3 |
| QITLNDLPVGR | 0 | 1 | 0 | 0 | 1 | 0 | 0 | 0 | 2 |
| QNQIAVDEIR | 2 | 2 | 0 | 0 | 0 | 0 | 0 | 0 | 2 |
| RLFEGNALLR | 0 | 2 | 1 | 0 | 1 | 0 | 0 | 0 | 3 |
| RLSEDYGVLK | 0 | 0 | 0 | 2 | 0 | 0 | 0 | 3 | 2 |
| SFAAVIQALDGEMR | 0 | 0 | 1 | 1 | 0 | 0 | 0 | 0 | 2 |
| SLLEGEGSSGGGGR | 9 | 0 | 0 | 0 | 0 | 3 | 0 | 0 | 2 |
| SNPEDQILYQTER | 0 | 0 | 0 | 0 | 1 | 1 | 0 | 0 | 2 |
| SQIHDIVLVGGSTR | 0 | 0 | 1 | 0 | 0 | 0 | 11 | 0 | 2 |
| SYELPDGQVITIGNER | 0 | 0 | 0 | 2 | 0 | 2 | 0 | 0 | 2 |
| TGAIVDVPVGEELLGR | 0 | 0 | 0 | 3 | 4 | 0 | 0 | 0 | 2 |
| THLAPYSDELR | 0 | 0 | 0 | 2 | 0 | 0 | 0 | 5 | 2 |
| TPAQYDASELK | 0 | 0 | 2 | 4 | 0 | 0 | 0 | 0 | 2 |
| VEYHFLSPYVSPK | 0 | 0 | 4 | 5 | 0 | 0 | 0 | 0 | 2 |
| VGGVQSLGGTGALR | 0 | 1 | 0 | 0 | 0 | 0 | 7 | 0 | 2 |
| VLSGDLGQLPTGIR | 0 | 0 | 0 | 0 | 1 | 0 | 3 | 0 | 2 |
| VTQWAEER | 1 | 1 | 0 | 0 | 0 | 0 | 0 | 0 | 2 |
| YLSYTLNPDLIR | 0 | 4 | 1 | 0 | 0 | 2 | 0 | 0 | 3 |

N: peptide hits of different samples

The 2nd to 9th columns are 8 human fetal liver samples marked by Pride accession numbers. The digit numbers in the 8 columns represent the number of all peptides from the corresponding gene mapped from the peptide in a row.
